# The role of EUS elastography-guided fine needle biopsy in the histological diagnosis of solid pancreatic lesions: a prospective exploratory study

**DOI:** 10.1038/s41598-022-21178-4

**Published:** 2022-10-05

**Authors:** Eizaburo Ohno, Hiroki Kawashima, Takuya Ishikawa, Yasuyuki Mizutani, Tadashi Iida, Ryo Nishio, Kota Uetsuki, Jun Yashika, Kenta Yamada, Masakatsu Yoshikawa, Noriaki Gibo, Toshinori Aoki, Kunio Kataoka, Hiroshi Mori, Yoshihisa Takada, Hironori Aoi, Hidekazu Takahashi, Takeshi Yamamura, Kazuhiro Furukawa, Masanao Nakamura, Yoshie Shimoyama, Yoshiki Hirooka, Mitsuhiro Fujishiro

**Affiliations:** 1 Department of Gastroenterology and Hepatology, Nagoya University Graduate School of Medicine, Nagoya University Hospital, 65 Tsurumai-cho, Showa-ku, Nagoya, Aichi 466-8550 Japan; 2 Department of Endoscopy, Nagoya University Hospital, Nagoya, Aichi Japan; 3 Department of Gastroenterology, Nakatsugawa Municipal Hospital, Gifu, Japan; 4 Department of Pathology and Laboratory Medicine, Nagoya University Graduate School of Medicine, Nagoya, Aichi Japan; 5 Department of Gastroenterology and Hepatology, Fujita Health University, Toyoake, Aichi Japan

**Keywords:** Cancer imaging, Gastrointestinal cancer, Imaging, Diagnostic markers

## Abstract

This study aimed to evaluate the feasibility and efficacy of Endoscopic ultrasound elastography-guided fine needle biopsy (EUS-EG-FNB) for the diagnosis of pancreatic mass lesions. EUS-EG images were classified into heterogeneous and homogeneous groups. For the heterogeneous group, EUS-FNB was separately performed in both hard areas and soft areas. Only samples obtained during the first two passes (hard/soft areas) were used to compare the diagnostic accuracy as well as the quality and quantity of the specimens. We investigated the association of EUS-EG findings using strain histogram analysis with the histological findings. Fifty-five patients were enrolled including 25 patients with heterogeneous group. The homogeneous group had significantly lower mean strain value (hard) lesions. The adequate sampling rates from hard and soft areas were 88 and 92%, respectively (*P* = 0.6374). Comparison of the diagnostic accuracy and the quality and quantity of the histological core between hard and soft areas showed no significant differences. In pancreatic adenocarcinoma cases, the proportion of fibrous stroma in the core tissue was significantly correlated with the elasticity of the region. (R^2^ = 0.1226: *P* = 0.0022) EUS-EG may reflect tissue composition in pancreatic tumors, however, EUS-EG did not affect either the quality and quantity of the tissues obtained.

*Clinical Trial Registry No*: UMIN-000033073.

## Introduction

Pancreatic ductal adenocarcinoma (PDAC) is a malignant disease that is associated with a poor prognosis, and the number of patients is increasing in Japan. Surgical resection is the only curative treatment strategy; however, less than 20% of PDAC patients are eligible for surgery at diagnosis, and most patients are already in an unresectable stage^1^. A well-known pathological feature of PDAC is the proliferation of cancer-associated fibroblasts (CAFs), excessive deposition of extracellular matrix (ECM) proteins produced by CAFs, and ECM remodeling in the stroma and intratumoral necrosis components^2,3^. Recently, precision medicine based on disease-specific biomarkers and genomic mutation profiles has become widespread, but a sufficient amount of core tissue sample is required to perform molecular and genetic profiling analysis^4–7^.

Endoscopic ultrasound (EUS)-fine needle biopsy (EUS-FNB) is a well-established endoscopic procedure of tissue acquisition for pancreatic and peripancreatic diseases^8,9^,^10^. On the other hand, in actual clinical settings, tissue samples collected by EUS-FNB are insufficient for molecular testing or genetic profile analysis^5,6^. Various tissue acquisition methods have been evaluated to determine the best approach for obtaining suitable tissue samples^8,11–13^.

We have previously reported the utility of EUS elastography (EUS-EG) in the differential diagnosis of pancreatic tumors^14–16^. PDAC is generally reported to exhibit higher elasticity than surrounding normal pancreatic parenchyma or other pancreatic lesions^17^. To date, the effect of the distribution of tissue elasticity in lesions of pancreatic tumors, especially for PDAC, on the tissue acquisition ability of EUS-FNB and the quality of obtained samples has not been investigated. Therefore, we conducted this prospective explorative study to elucidate the feasibility of performing EUS-EG guided fine needle biopsy (EUS-EG-FNB) in these patients and to determine the effect of the differences in tissue elasticity observed by EUS-EG on both the amount and quality of core tissue as well as the histological diagnostic ability for pancreatic mass lesions.

## Methods

### Study design and ethical consideration

This was a single-center, prospective exploratory study comparing the tissue acquisition rate of EUS-EG-FNB between hard areas and soft areas in pancreatic mass lesions. This study was conducted from November 2018 to April 2021 at Nagoya University Hospital. This study was approved by the Institutional Review Board in Nagoya University Hospital (IRB #2018–0217), registered with the University Hospital Medical Information Network (UMIN-CTR No. UMIN-000033073 Registered date01/07/2018) and conducted according to the provisions of the Declarations of Helsinki.

### Patients

Patients undergoing EUS-FNB for pancreatic solid mass lesions were candidates for this study. We obtained written informed consent from patients aged 20 years or older who required pathological diagnosis by EUS-FNB and participated in the study before EUS procedures. The exclusion criteria were (1) patients whose pancreatic mass could not be visualized by EUS, (2) patients with pancreatic cystic tumors, (3) patients younger than 20 years old, (4) patients at high risk of bleeding, (5) pregnant patients and (6) patients who refused to participate in the study.

### Procedural technique

All procedures were performed using a linear-array echoendoscope by experienced endoscopists who were well trained in pancreaticobiliary EUS. Prior to performing EUS-FNB, detailed observation of the pancreatic mass lesion site by EUS was performed. In EUS-EG, the ROI was set to include the entire tumor at the position where the pancreatic mass lesion could be visualized on the maximum diameter on EUS images. We used the strain elastography (SE) method with strain histogram (SH) analysis to measure and quantify the tissue elasticity. EUS-EG images were recorded as moving images, and high-quality still images in which the signal was drawn over the entire ROI were recorded. After evaluating the lesions by EUS-EG, EUS-FNB was performed. We used a GF-UCT260 linear-array echoendoscope (Olympus Co., Tokyo, Japan) and an ARIETTA 850 (Fujifilm healthcare corp, Tokyo, Japan) and EU-ME2 premier plus (Olympus Co., Tokyo, Japan) as ultrasound processors in this study.

A 22-gauge Franseen needle (Acquire™, BostonScientific Japan) was used to perform EUS-FNB in all patients. At the time of EUS-FNB, the puncture was performed with the stylet slightly removed, and the tissue of the target area was collected by pushing out the stylet before collecting tissue to avoid tissue contamination. After the needle was inserted into the pancreatic mass lesion, the needle was moved back and forth 15 times for sample acquisition using the slow-pull method. The collected specimens were immediately placed in formalin solution to prevent drying of the tissue and sent for histology. The sample was not split for cytology or cell block.

**Figure 1:**
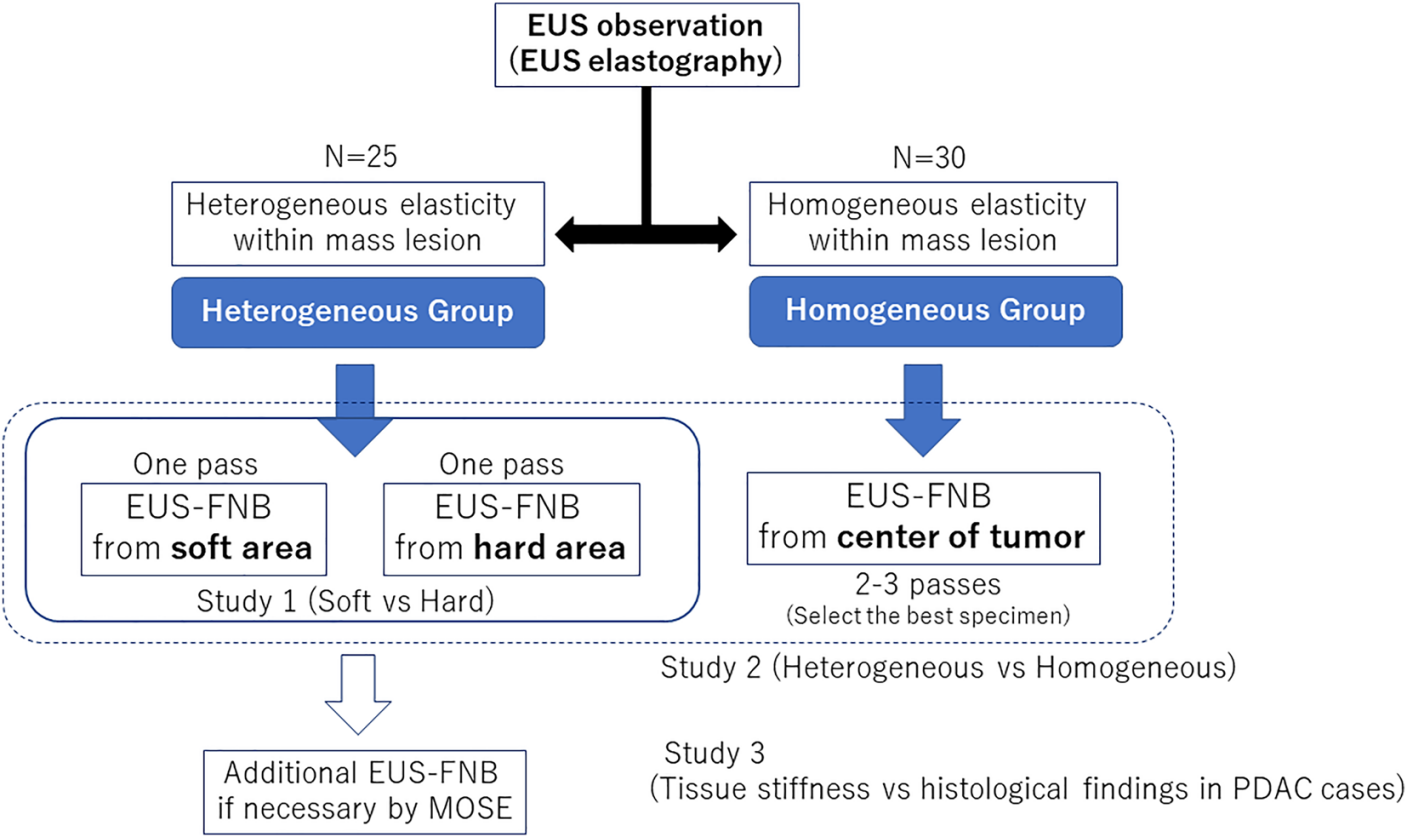
Flow diagram of study participants.

For patients in which the tissue elasticity showed heterogeneity in the mass lesion by EUS-EG (heterogeneous group), one sample each was obtained from the high elasticity region (hard area: presented in blue) and the low elasticity region (soft area: presented in red to green) in the mass lesion. For patients in which the tissue elasticity in the mass lesion was uniform (homogeneous group), a sample was collected from the center of the tumor. The evaluation of heterogeneity based on EUS-EG image findings was diagnosed when the reproducibility was confirmed by at least a 10-s movie and 3 or more still images. For the puncture order for the hard area/soft area, sampling was performed in the region closer to the EUS probe first. When a sufficient amount of tissue was not obtained from the first two passes, additional punctures were performed until a sufficient amount of tissue was obtained based on macroscopic on-site evaluation (MOSE). Specimens that underwent additional puncture were excluded from the evaluation of this study. For the homogeneous group, 2 or 3 samples were obtained from the lesion, and the best sample was used for histological evaluation and comparison with the heterogeneous group (Fig. 1).

### Analysis of EUS-elastography images

SH analysis was performed on the EUS-EG images to quantify the tissue elasticity of pancreatic mass lesions. The method used for SH analysis was performed as previously reported^15^. The mean strain value (MSV) was measured for the "hard area" and "soft area" within the ROI and the "whole lesion" in which the entire tumor was set as the ROI in the maximum cross section of the pancreatic mass. A lower mean value indicates greater elasticity, and it has been reported to be a useful method for analysis with the strain method. We extracted three representative EUS-EG images at the puncture sites. The median of three MSVs of each patient was defined as the representative module of the target patient. Histogram analysis was performed using Elasto_ver. 15.1 (Hitachi-Aloka Medical, Ltd., Tokyo, Japan) offline to evaluate the EG images from the two systems using the same method.

### Classification of EUS-elastography images

**Figure 2:**
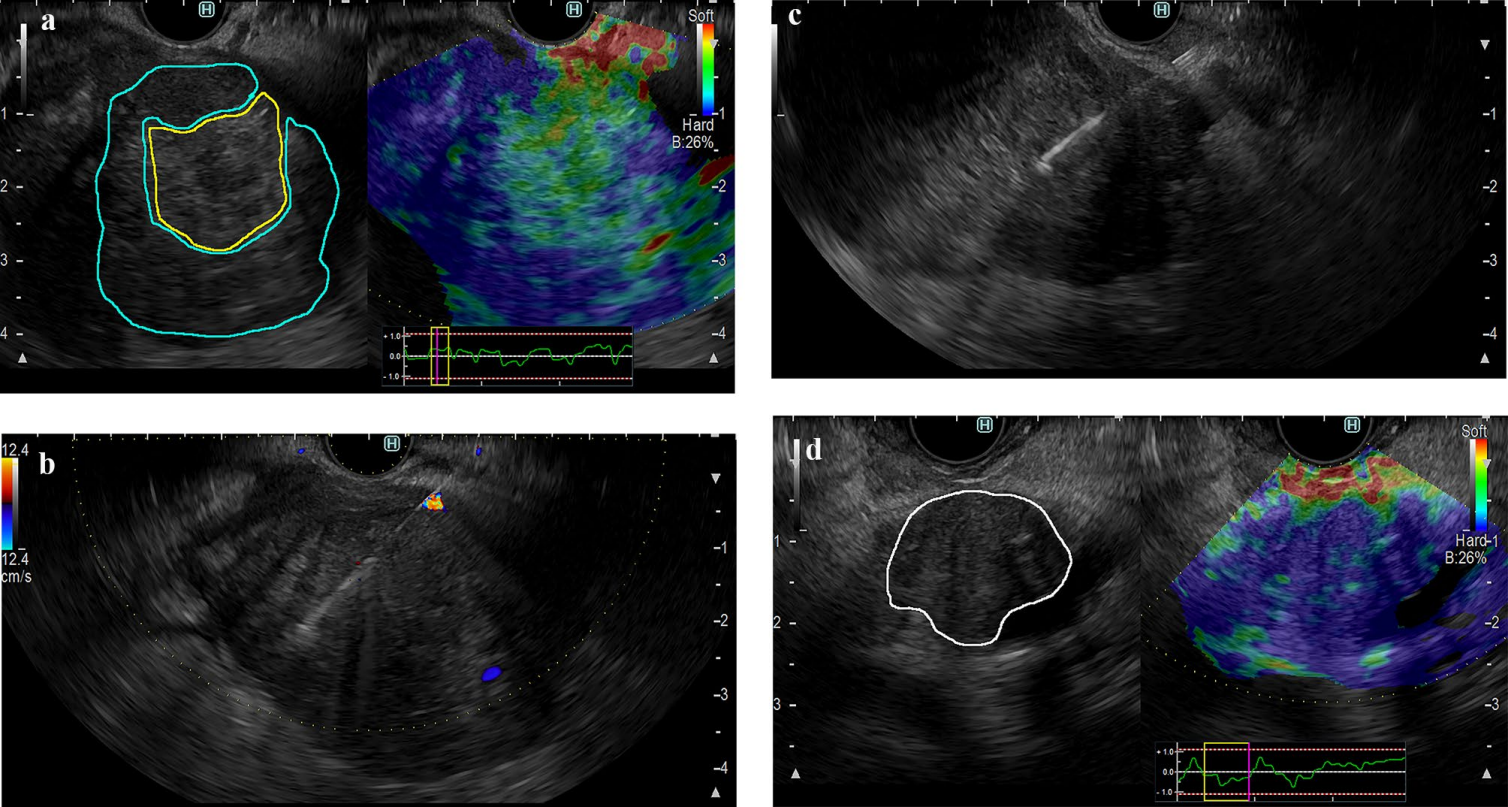
Representative EUS elastography findings of pancreatic solid mass lesions. The yellow surrounding line shows the soft areas, and the blue surrounding line shows the hard areas. In the homogeneous group, the white surrounding line shows the contours of the tumor. (**a**) A patient in the heterogeneous group, with pancreatic ductal adenocarcinoma. The mean strain values in the whole lesion, soft area and hard area were 68.0, 122.2 and 31.4, respectively. (**b**) EUS-FNB to the hard area in the patient shown in Fig. 3a. (**c**) EUS-FNB to the soft area in the patient shown in Fig. 3a. (**d**) A patient in the homogeneous group, with pancreatic ductal adenocarcinoma. The mean strain value in the whole lesion was 41.8.

We hypothesized that the necrotic tissue may be displayed as the soft region, which would be in the central part of the lesion. Therefore, we classified the location of the soft lesion to compare EG findings and histological features. The findings in the pancreatic mass by EUS-EG were classified into less than 10, 11–30%, 31–50, and 51% or more by the soft area occupied rate in the mass lesion. EUS-EG was used to identify patients in which the inside of the mass could be divided into hard and soft areas, defined as the heterogeneous group, and those with uniform hardness in the mass (hardness unevenness of less than 10%), defined as the homogeneous group (Fig. 2).

### Preparation and review of specimens obtained by EUS-FNB

All samples obtained by EUS-FNB were subjected to histological analysis. The histological assessment was performed by an experienced pathologist at Nagoya University Hospital (Y.S.) based on the cellularity scoring system^18^. The samples were submitted such that the pathologist was blinded to the tissue elasticity results for each lesion. The cellularity of the samples was scored from 0 to 5. The adequacy of the samples assessed for histological diagnosis was scored as follows: a score of 0–2 was defined as “inadequate”, and a score of 3–5 was defined as “adequate”. “Malignancy” was defined as either positive or suspected to be malignant, and “benign” was defined as either negative or atypical. To evaluate the amount of core tissues obtained for each specimen, the total length of the core tissue core was measured under a photomicroscope using imaging software (CellSense; Olympus Co. Ltd., Tokyo, Japan). The specimen was captured under low magnification so that the whole specimen was included in a single image, and the total length of the core tissues was measured manually^19^. In addition to the qualitative diagnosis (tissue score) and quantitative diagnosis (core tissue length) of EUS-FNB, the proportion of fibrous stroma in the core tissue was evaluated to compare the tissue elasticity with the characteristics of the collected tissue (Supplementary Figs. 1–3). Histopathological evaluation was performed using HE-stained specimens. The pathologist assessed the proportion of fibrous stroma as measured the fibrotic tissue length and core tissue length in units of 10% by using images in CellSense.

### Final diagnosis

Patients were diagnosed with malignant disease if metastatic lesions were identified during imaging examinations, if there were signs of disease progression, and/or if malignant EUS-FNB results were obtained. Patients were diagnosed with benign disease if they had a nonresected mass that did not display imaging features of malignancy during at least 6-month follow-up or if they had EUS-FNB results suggestive of a benign lesion with additional needle passes.

### Outcome measures

The primary endpoint of this study was a comparison of histological diagnostic ability of EUS-EG-FNB between the soft area and the hard area within the pancreatic mass lesions (Study 1). The secondary endpoints were as follows: (1) association of heterogeneity in pancreatic mass lesions with histopathological diagnosis (Study 2), and (2) comparison of EUS-EG findings and histological findings in PDAC patients (Study (3). Any adverse events were recorded and compared according to the lexicon for endoscopic adverse events advocated by the American Society of Gastrointestinal Endoscopy^20^. Quantitative analysis of EUS-EG was blindly analyzed for clinical and pathological diagnosis. Similarly, pathological assessments were performed blinding to EUS-EG findings.

### Statistical analysis and sample size calculation

The accuracy rates in EUS-FNA have been reported to range from 65 to 90%. We hypothesized that the necrotic tissue in the tumor may exhibit characteristics as a soft area. Kamata et al. reported that the sensitivity of EUS-FNA for lesions with and without an avascular area in CH-EUS was 72 and 94%, respectively^21^. Since the avascular area in CH-EUS may reflect intratumoral necrosis, we referred to this result to determine the sample size and the classification of the soft area location in this study. Assuming that the detection rate of tumor tissue when collected from the hard area by elastography is 90% and the detection rate of tumor tissue when collected from the soft area is 65%, the difference in the score is the standard deviation. When it corresponds to 60%, that is, when the effect size of Cohen is a medium effect size (d = 0.6), a total of 42 patients are needed to detect the difference under the conditions of significance level 0.05 and power 80%. The target number of patients was set at 45 at the time of planning the study. In this study, the sampling, sensitivity, and specificity rates were analyzed using the McNemar test. Descriptive statistics are expressed as the median and interquartile range (IQR). Qualitative variables were compared by χ2 tests, and quantitative variables were compared using the Mann–Whitney U test. Data were statistically analyzed using JMP Pro version 12 (SAS Institute, Inc., Cary, NC, USA).

## Results

Written informed consent was obtained from 55 patients with pancreatic mass lesions during the study period. However, unexpectedly few patients exhibited elastic heterogeneity in pancreatic mass lesions. An interim analysis was performed when 25 patients were enrolled who exhibited sufficient heterogeneity in the tissue elasticity in pancreatic mass lesions that separate punctures could be performed. The analysis results of the study are presented for the heterogeneous group and the homogeneous group, in which the tissue elasticity was uniform (the rate of soft area was less than 10%) depicted by EUS-EG. The baseline characteristics of the patients and final pathological diagnosis are summarized in Table 1. There were no adverse events associated with the EUS-EG-FNB procedure.

**Table 1:** Clinical Characteristics and final diagnosis of patients.

Variable		Total	Heterogeneous	Homogeneous	
		n = 55	n = 25	n = 30	*P* value
Sex	Male/Female	30/25	15/10	15/15	0.4583
Age	Median (IQR)	68(62–76)	66(61.5–73.5)	72(61.5–78.3)	0.6888
Location	Ph: Pb: Pt	19:19:7	4:8:13	15:11:4	0.0036
Tumor size	Median (IQR)	30(22–36)	35(25–50)	24.5(20–30)	< 0.0001
NCCN resectability	R:BR: UR-LA:UR-M	11:7:7:28	2:1:4:17	8:6:3:11	0.0593
Diabetes	( +) (%)	18(32.7)	9(36)	9(30)	0.6368
Obstructive jaundice	( +) (%)	13(23.6)	4(16)	9(30)	0.2237
Symptoms	( +) (%)	42(76.4)	22(88)	20(66.7)	0.0637
Serum CA19-9	Median (IQR) (U/mL)	308(33–1636)	339(64.5–10,120)	213(27.8–1277)	0.2588
Serum CEA	Median (IQR) (ng/mL)	4(2.3–12.3)	3.8(2.2–18.2)	4(2.3–10.4)	0.8638
Number of pass	Median(range)		2(2–3)	2(2–2)	0.0044
**Final diagnosis**					
PDAC		50	21	29	0.3793
MFP		2	2	0	
Neuroendocrine tumor		1	0	1	
Acinar cell carcinoma		1	1	0	
Anaplastic carcinoma		1	1	0	

*NCCN* National Comprehensive Cancer Network.

*R* resectable, *BR* borderline resectable, *UR-LA* unresectable with locally advanced, *UR-M* unresectable with metastasis.

CA19-9: Carbohydrate Antigen 19–9.

*CEA* Carcinoembryonic Antigen.

*PDAC* Pancreatic Ductal Adenocarcinoma.

*MFP* Mass Forming Pancreatitis.

### EUS-elastography findings

Table 2 shows the EUS-EG findings of all participating patients. The MSV of EUS-EG including the whole tumor was significantly lower in the homogeneous group than in the heterogeneous group (*P* < 0.0001). There was also a significant difference in the MSV between the hard and soft regions in the heterogeneous group (*P* < 0.0001). In addition, the region occupied by the soft area in the lesion was significantly different between the heterogeneous group and the homogeneous group.

**Table 2:** EUS findings.

Variable		Heterogeneous		Homogeneous	*P* value
MSV (whole)	Median (IQR)	50.5(38.9–66.7)		28.4(18.9–31.4)	< 0.0001
		Hard	Soft		
MSV (area)	Median (IQR)	24.4(15.8–30.5)	100.9(67.7–122.0)	-	< 0.0001
Proportion of soft area		n		n	
	~ 10%	0		27	< 0.0001
	11 ~ 30%	3		0	
	31 ~ 50%	15		0	
	51% ~	7		3	

*MSV* the mean strain value.

### Comparison of histological diagnostic ability of EUS-EG-FNB between the soft area and the hard area within the pancreatic mass lesions (Study 1)

Table 3 shows the results of histological comparison between tissue elasticity as the primary endpoint and the qualitative evaluation of the pathological tissue. No significant difference was found in the adequacy of histological samples from the hard and soft areas (*P* = 0.7744). In addition, no significant difference was observed between the hard and soft areas in the measurement of the collected core tissue length (*P* = 0.7718). The diagnostic sensitivity based on the elasticity of the lesion was 92% in the hard area and 84% in the soft area, with no significant difference observed (*P* = 0.3841).

**Table 3:** Pathological findings.

	Heterogeneous		Homogeneous	*P* value
	Hard area	Soft area		
**Qualitative evaluation**				
Score 0	0	0	0	0.7744*
1	2	2	0	
2	1	0	3	
3	0	0	2	
4	4	5	8	
5	18	18	17	
Score Median(IQR)	5 (4–5)	5(4–5)	5(4–5)	N.S
Adequate sampling rate(%)	88%	92%	90%	0.6374* 0.7800** 0.8268***
Sensitivity (95%CI)	92%(83–95)	84%(68–98)	90%(81–96)	0.3841* 0.7973** 0.5062***
Specificity	100%	100%	100%	N.S
**Quantitative evaluation**				
Length of core (mm) All cases	6.9(3.8–8.3)	6.3(3.9–10.1)	5.26(3.8–7.7)	0.7718* 0.4017** 0.2473***
	Hard area	Soft area	Homogeneous	
Proportion of fibrous stroma (%)#	60(50–80)	60(20–80)	70(50–80)	0.3309

*Hard versus Soft,

**Hard versus Homogeneous,

***Soft versus Homogeneous.

^#^Proportion of fibrous stroma in the core tissue.

### Association of heterogeneity in pancreatic mass lesions with histopathological diagnosis (Study 2)

**Figure 3:**
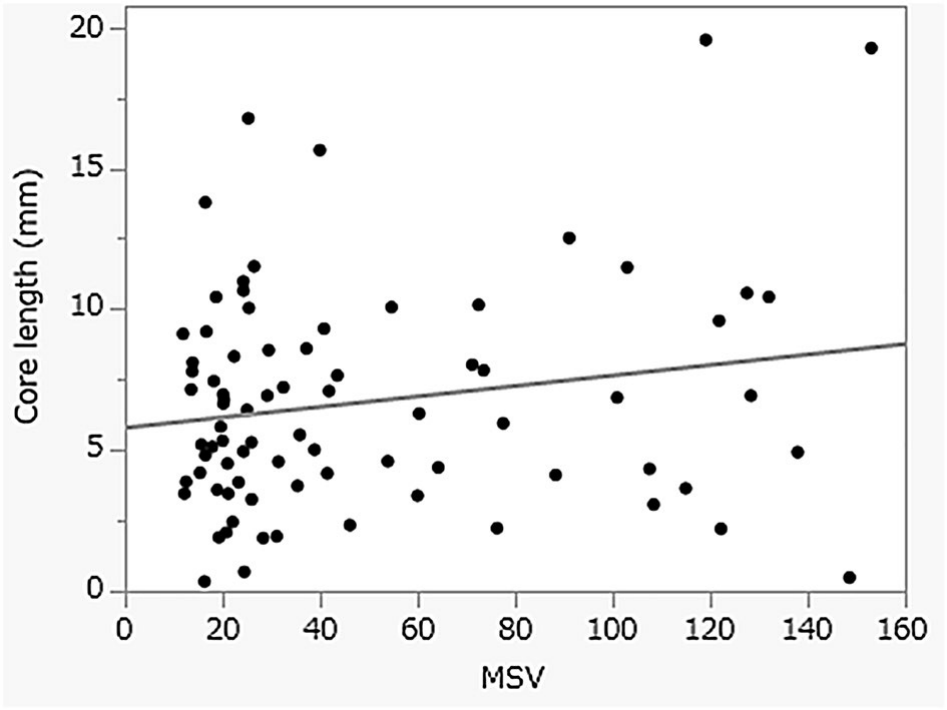
The correlation between the mean strain value at the puncture site and the core tissue length. (R^2^ = 0.0344: *P* = 0.0993).

The histological findings of 30 pancreatic mass lesions in the homogeneous group were compared with those of 25 lesions in the heterogeneous group. There was no statistically significant difference in the quality of collected tissue (histological score), diagnostic sensitivity, advanced sampling rate, or core tissue length observed among the hard areas, soft areas, and homogeneous areas identified by EUS-EG. In addition, no significant correlation was found between the MSV at the puncture area and the core tissue length in all target patients (R^2^ = 0.0344: *P* = 0.0993) (Fig. 3).

### Comparison of EUS-EG findings and histological findings in PDAC patients (Study 3)

**Figure 4:**
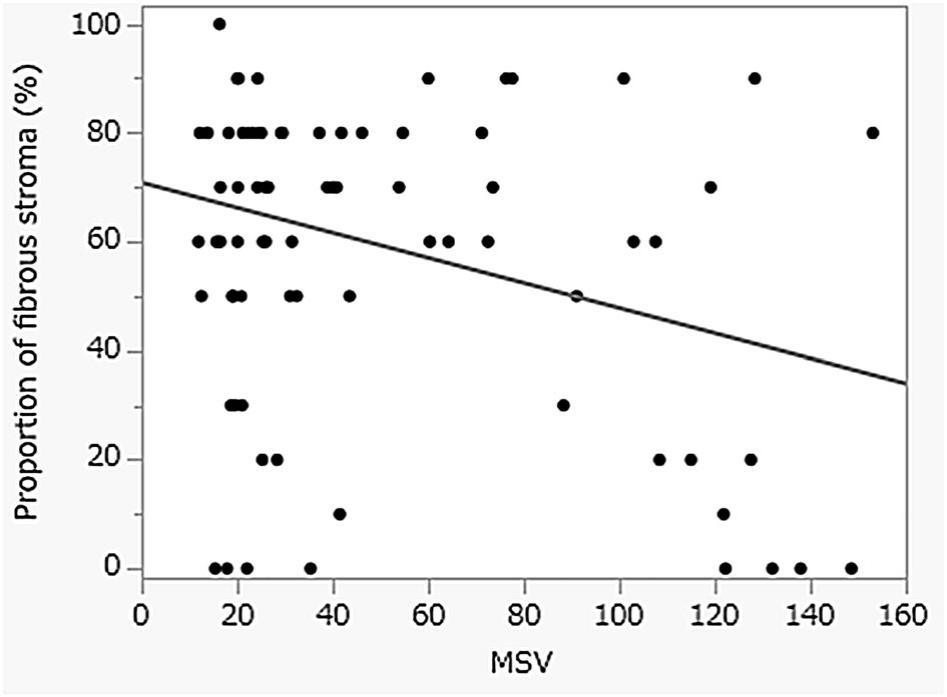
The correlation between the mean strain value at the puncture site and the proportion of fibrous stroma in the core tissue in cases of pancreatic ductal adenocarcinoma. (R^2^ = 0.1226: *P* = 0.0022).

In terms of comparing the tissue elasticity and the histological characteristics of PDAC patients, the proportion of the fibrous stroma in the core tissue was a significantly negatively correlated with MSV. (R^2^ = 0.1226: *P* = 0.0022) This result indicated that the harder area in PDAC had a dominant fibrous stroma (Fig. 4).

## Discussion

In this study, we attempted to investigate the association between the difference in elasticity measured by EUS-EG in a solid pancreatic mass lesion and the histological diagnosis of EUS-FNB specimens. A significant difference was found in the elasticity of the whole tumor between the heterogeneous group and the homogeneous group. Interestingly, there was a significant correlation between the proportion of fibrous stroma in the core tissue and the tissue hardness. On the other hand, there was no significant difference in the amount and quality of the collected tissue in the puncture by tissue hardness, and the heterogeneity of elasticity in the pancreatic solid tumor by EUS-EG did not contribute to the diagnostic ability of EUS-FNB.

In PDAC, which is a refractory cancer, 90% of the total tumor volume is composed of proliferative connective tissue fibroblasts, ECM components such as collagen and CAFs, and vasculature, and the increased tissue elasticity induced by these extracellular matrix components further stimulates cancer cells. In typical PDAC tissue, abundant fibroblasts surrounding the cancer cells establish an ischemic tissue environment, resulting in increased resistance to anticancer drugs^3,22–24^.

Various methods to clinically measure tissue elasticity in the human body are being investigated. Ultrasound elastography is a technology developed in Japan in 2003 that noninvasively measures and visualizes tissue elasticity^25^. PDAC tissue generally showed higher elasticity than the normal pancreatic parenchyma and other pancreatic diseases. However, the image of SE represents relative stiffness inside the ROI, the quantification of EG images is rather complicated, and the relationship between tissue elasticity and pathological findings within the same disease has not yet been fully elucidated^15,26–28^. In 2020, Shi et al. reported that the prognosis of PDAC patients with high tissue elasticity (hard tumors) was significantly poor and that tissue elasticity was associated with the amount of fibrotic changes in PDAC^29^.

As our preliminary hypothesis, it was predicted that low-elasticity areas reflect tissue necrosis and that the diagnostic ability would decrease for tissues obtained from low-elasticity areas. However, no difference was observed in the amount of tissue obtained by the commonly used 22 Gauge EUS-FNB needle, which can affect the quality of the collected tissue as well as the diagnostic ability. This may be because the tissues collected during the EUS-FNB included not only obtained from the same elasticity region through one needle pass.

Contrast harmonic EUS (CH-EUS) is another method of image enhancement software in EUS^29^. CH-EUS has been reported to be useful for characterizing various types of pancreatic diseases, including PDAC, with excellent contrast resolution^30–32^. In some studies, EUS-FNB was performed under CH-EUS^21,33–35^. Itonaga et al. reported that the rate of adequate sampling with CH-EUS-guided FNB was superior to that with conventional EUS-FNB in a prospective study^13^. Image-enhanced EUS such as EUS-EG and CH-EUS may reflect histological features in PDAC and pancreatic tumors, including histological heterogeneity, as in this study.

There are several limitations to this study. First, this was a preliminary prospective single-center study with a small number of patients. We hypothesized that the necrotic tissue in the tumor may exhibit characteristics as a soft area. However, the elasticity of the pancreatic tumor may be comprised not only by the intratumoral necrosis but also by fibrous stroma or cancer cellularity. The number of patients required for examination was estimated to be 42, but the proportion of patients with heterogeneity in elasticity by EUS-EG to allow for separate punctures to be obtained was much lower than expected; for this reason, we investigated 25 patients as an intermediate analysis. In this study, the number of cases was estimated from the results of the CH-EUS study to compare the diagnostic ability of FUS-FNB from each elasticity in the tumor. However, due to differences in diagnostic software, this criterion may have been inappropriate. To our knowledge, there is no prospective study that compared the histological findings and tissue elasticity by EUS-EG, and it may be necessary to recalculate the number of cases based on the results of this study or the results of retrospective studies. Second, the pathological evaluation was performed by a single expert pathologist mainly with HE-staining findings. From the viewpoint of reproducibility, the evaluation may be better to be performed by multiple pathologists. Third, more than 90% of the patients in this study had PDAC. A diagnosis of PDAC by EUS-FNA/B may be relatively easy to make because the proof of PDAC cells, including cytological assessment. On the other hand, the proportion of fibrous stroma in the core tissue was significantly correlated with the tissue elasticity in patients with PDAC. These results suggest that the tissue elasticity of pancreatic cancer is related to the degree of fibrosis. However, in this study, molecular testing or genetic profile analysis such as oncogene panel tests was not performed. The proportion of cancer cells in the biopsy tissue is associated with the analysis success rate of the oncogene panel test. In the future, it will be necessary to evaluate the correlation between tissue elasticity of pancreatic cancer and the appropriateness of the tissue for genetic testing.

In conclusion, approximately 40% of pancreatic masses subjected EUS-FNB show uneven tissue hardness in tumors with EUS-EG. Although the tumor hardness as determined by EUS-EG correlated with the proportion of fibrous stroma, the differences in tissue elasticity observed on EUS-EG did not affect the pathological diagnosis by EUS-FNB or the quality of the obtained tissue.

## Supplementary Information

Supplementary Information 1.

Supplementary Information 2.

Supplementary Information 3.

Supplementary Information 4.

Supplementary Information 5.

Supplementary Information 6.

## Data Availability

The datasets used and/or analysed during the current study available from the corresponding author on reasonable request.
