# Supplementary material for: The role of EUS elastography-guided fine needle biopsy in the histological diagnosis of solid pancreatic lesions: a prospective exploratory study
Source: Sci Rep. 2022 Oct 5;12:16603. doi: 10.1038/s41598-022-21178-4 (PMC9535001; doi:10.1038/s41598-022-21178-4)
Supplement: Supplementary file 1 — Supplementary Information 1. [file 41598_2022_21178_MOESM1_ESM.docx]

**Figure Legends of supplementary figures**

Supplementary figure 1

A representative pancreatic adenocarcinoma case of EUS-FNB.

The length of core tissue was measured using CellSence.

Supplementary figure 2a

A representative specimen obtained from the hard area.

The length of core tissue was 19.26mm and the proportion of fibrous stroma was 80%.

Supplementary figure 2b

EUS-EG image. EUS-FNB was performed from the hard area in the tumor.

The MSV of puncture site was 39.936.

Supplementary figure 3a

A representative specimen obtained from the soft area.

The length of core tissue was 10.57mm and the proportion of fibrous stroma was 20%.

Supplementary figure 3b

EUS-EG image. EUS-FNB was performed from the soft area in the tumor.

The MSV of puncture site was 127.54.
