# Supplementary figures and images for: The role of EUS elastography-guided fine needle biopsy in the histological diagnosis of solid pancreatic lesions: a prospective exploratory study
Source: Sci Rep. 2022 Oct 5;12:16603. doi: 10.1038/s41598-022-21178-4 (PMC9535001; doi:10.1038/s41598-022-21178-4)

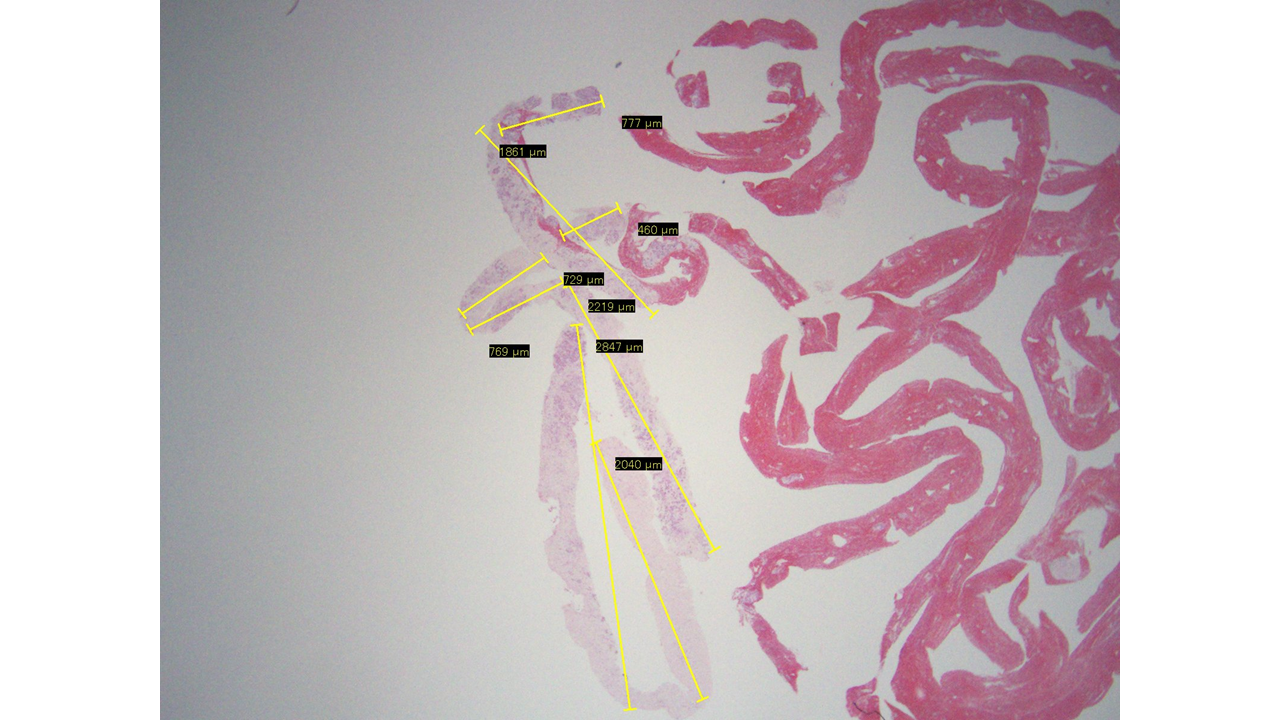

Supplement: Supplementary file 2 — Supplementary Information 2. [file 41598_2022_21178_MOESM2_ESM.tif]

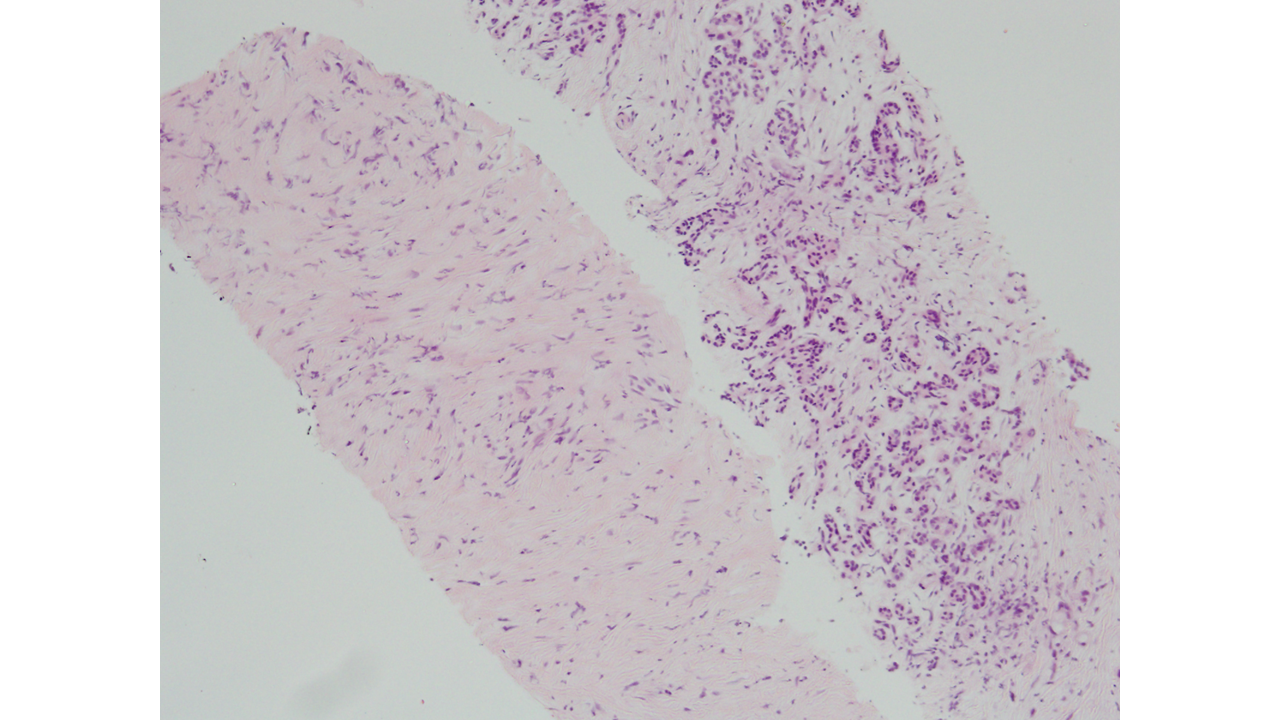

Supplement: Supplementary file 3 — Supplementary Information 3. [file 41598_2022_21178_MOESM3_ESM.tif]

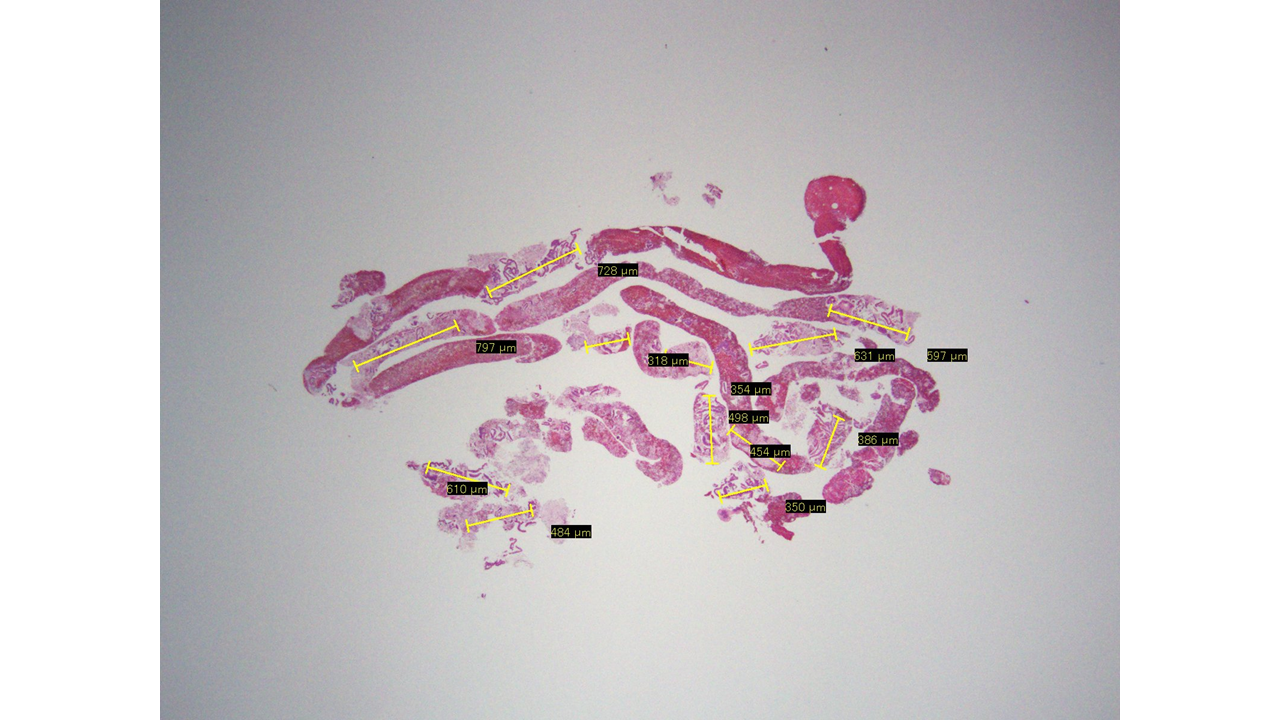

Supplement: Supplementary file 4 — Supplementary Information 4. [file 41598_2022_21178_MOESM4_ESM.tif]

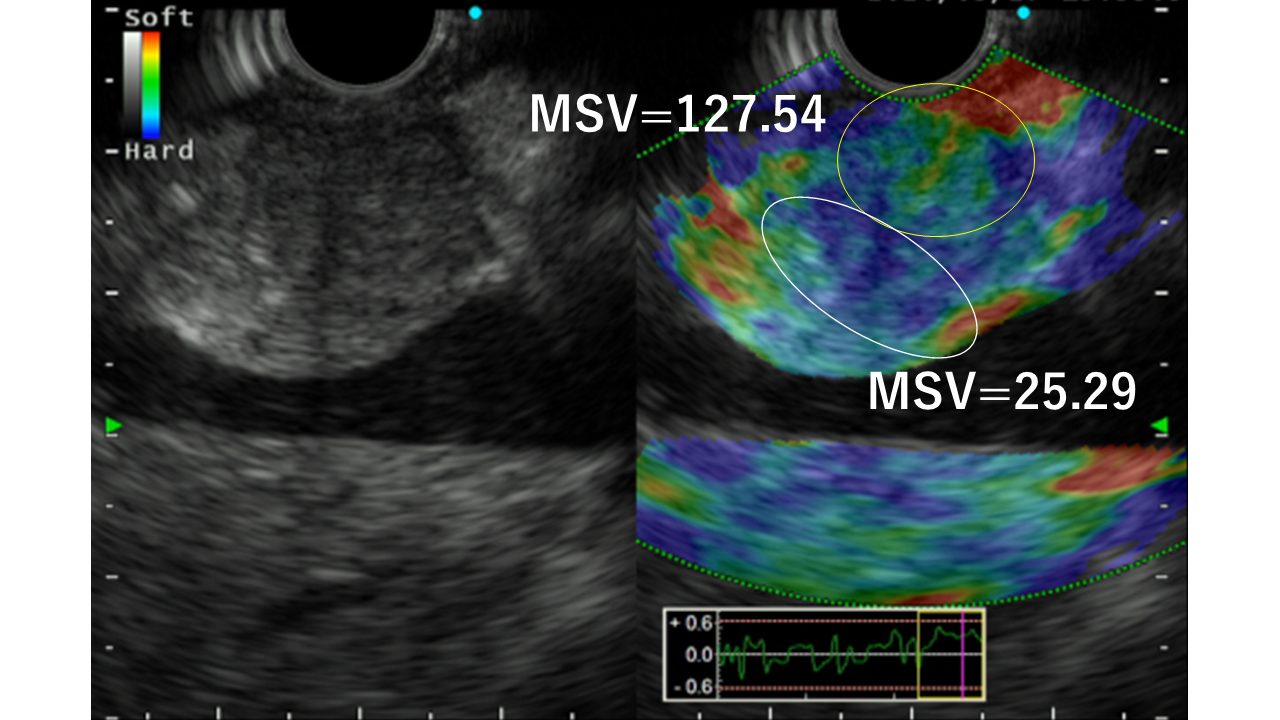

Supplement: Supplementary file 5 — Supplementary Information 5. [file 41598_2022_21178_MOESM5_ESM.tif]

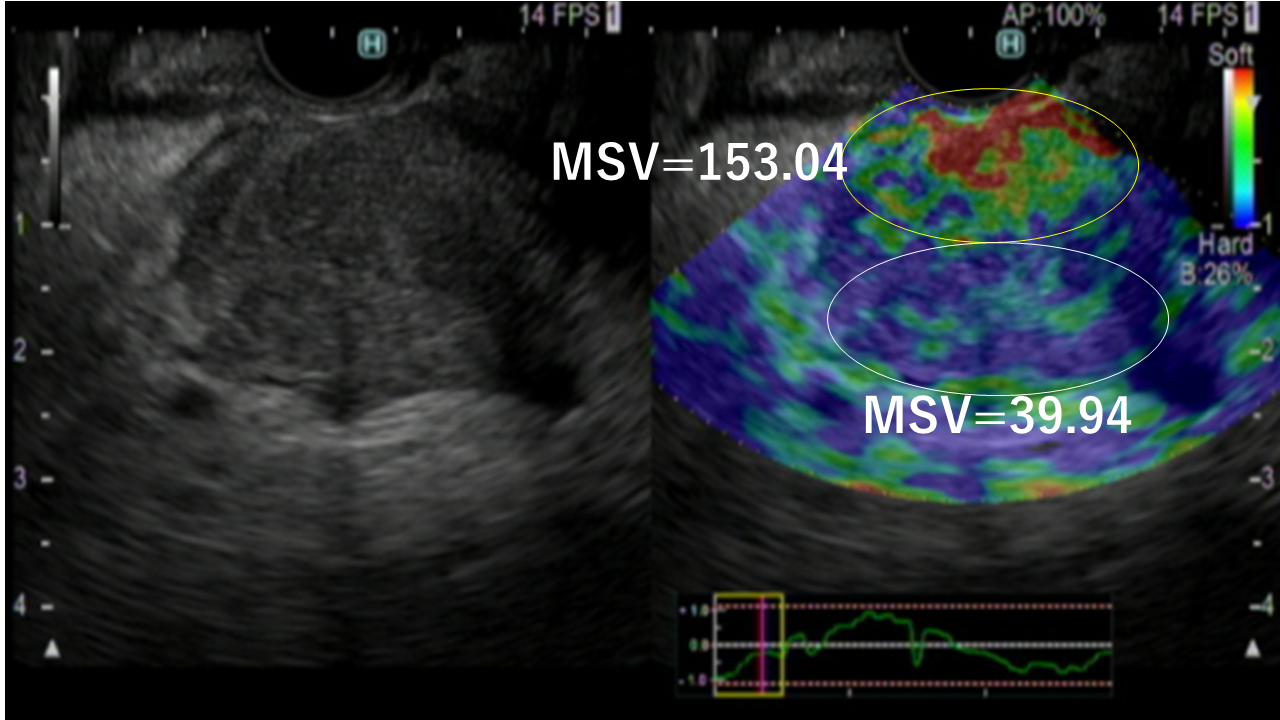

Supplement: Supplementary file 6 — Supplementary Information 6. [file 41598_2022_21178_MOESM6_ESM.tif]
